# *Listeria monocytogenes* Survival on Peaches and Nectarines under Conditions Simulating Commercial Stone-Fruit Packinghouse Operations

**DOI:** 10.3390/ijerph18179174

**Published:** 2021-08-31

**Authors:** Deepa Kuttappan, Muhammed S. Muyyarikkandy, Elza Mathew, Mary Anne Amalaradjou

**Affiliations:** 1 Department of Animal Science, University of Connecticut, Storrs, CT 06269, USA; deepa.kuttappan@uconn.edu (D.K.); mmuyyar@ncsu.edu (M.S.M.); elza.mathew@umassmed.edu (E.M.); 2 Department of Pathobiology and Population Health, College of Veterinary Medicine, North Carolina State University, Raleigh, NC 27607, USA; 3 Department of Neurological Surgery, University of Massachusetts Medical School, Worcester, MA 01605, USA

**Keywords:** *Listeria monocytogenes*, survival, stone fruits, packinghouse operations

## Abstract

Recent recalls of stone fruit due to potential *Listeria* contamination and associated foodborne outbreaks highlight the risk for pathogen transmission through stone-fruit consumption. Particularly, surface contamination of fruits increases the risk for cross-contamination of produce during processing and storage. This highlights the need for quality control in stone fruits intended for consumption. To develop effective food safety practices, it is essential to determine the critical factors during stone-fruit processing that influence *Listeria* survival. Therefore, this study evaluated the ability of *Listeria* to survive on peaches and nectarines under simulated stone-fruit loading and staging, waxing and fungicide application and storage conditions. The results of our study indicate that current stone-fruit handling conditions do not favor *Listeria* growth. However, once fruit is contaminated, *Listeria* can survive on the fruit surface in significant numbers under current processing conditions. Therefore, there is a need to develop and implement preventive controls at the stone-fruit packinghouse to prevent *Listeria* contamination and deter pathogen persistence.

## 1. Introduction

With increasing consumer awareness and demand for maintaining a healthier lifestyle, the consumption of fresh fruits and vegetables has increased over the last few decades [1]. However, the increased dietary incorporation of fresh produce has been linked to an increase in the incidence of produce-related foodborne outbreaks [2,3,4]. Human pathogens associated with produce include *Salmonella*, *E. coli* O157, *Listeria monocytogenes*, *Bacillus cereus*, *Aeromonas* and *Shigella* spp., Norovirus, Hepatitis A and Cyclospora [3,5]. Of these, *Salmonella, E. coli* O157:H7 and *L. monocytogenes* are the leading bacterial causes of produce-related foodborne illnesses [3,6]. Although primarily associated with dairy and RTE foods, *Listeria* has been increasingly implicated in different produce outbreaks and recalls including butternut squash, cauliflower, zucchini, broccoli, tomatoes, snap peas, cantaloupes, lettuce, sprouts and stone fruits [1,7,8,9,10,11,12].

Stone fruit, including peaches, nectarines, plums and pluots are generally considered as low risk fruit for foodborne illnesses [13]. However, in 2014, detection of *Listeria* led to a national recall of stone fruits. Furthermore, this recall was associated with a multistate outbreak that resulted in one fatality [10,14,15]. This first reported link between human listeriosis and stone fruit highlights a potentially new food vehicle in the transmission of *Listeria monocytogenes*. In addition, this outbreak also demonstrates the pathogen’s ability to persist and survive on stone fruits through the handling, storage and transportation chain. Pathogen presence on the fruit’s surface indicates that inadequate hygienic conditions were employed during harvesting, processing and/or transportation. This emphasizes the need for quality control in stone fruits intended for consumption.

The contamination of stone fruit can occur at any point along the production continuum. In the pre-harvest environment, fruit can get contaminated from contact with soil, water and animal manure [13,16,17]. In this regard, *L. monocytogenes* has been isolated from soil, waterways and vegetation, where it exists as a saprophyte [18,19,20,21,22]. Besides these sources, *Listeria* is known to be associated with the gut microflora of domestic and wild animals and shed in feces [23,24]. Therefore, use of improperly composted manure, wildlife access to the orchard and animal rearing adjoining the produce field can act as source of contamination to the stone fruit [13,25,26,27]. Once harvested, fruit can get contaminated during processing and transportation from food-contact surfaces, including equipment and handlers [26,28,29]. Moreover, given that fruit are consumed as raw or minimally processed foods, pathogen control on produce becomes more challenging. Further, heightened consumer demand for fresh produce has also led to the handling of large quantities of produce which are obtained from different producers, resulting in the pooling of raw products and extensive handling by several types of equipment and individuals. These factors further increase the risk for cross-contamination of produce during processing and storage [16,30]. Therefore, incorporation of good management practices in the post-harvest environment is critical to promoting produce safety [31].

In the post-harvest setting, survival of *L. monocytogenes* on produce is primarily influenced by environmental parameters, including temperature and relative humidity encountered in the packinghouse and during distribution [8,15,32,33,34,35,36]. Besides fruit handling and storage conditions, fruit waxing and fungicide treatments are frequently employed in stone-fruit packinghouses [37]. As with temperature and RH, application of wax and fungicide can influence pathogen persistence and contamination on produce [32,38,39,40,41,42,43]. Another important factor that influences pathogen survival is the produce type [44]. Each produce type has a unique combination of compositional and physical characteristics that require specific growth conditions, harvesting protocols, processing practices and storage conditions [38]. So best management practices and preventive controls are highly produce-specific. Therefore, to develop effective food safety practices, it is essential that each produce is evaluated appropriately under conditions that are applicable to its processing and storage. Hence, the objective of this study was to determine the influence of stone-fruit handling, processing and storage conditions on *Listeria* survival on peaches and nectarines. Ambient conditions (temperature and RH), length of storage, fruit finish and fungicides used in the study are based on discussions with the California Fresh Fruit Association and adapted from the food safety guidelines for fresh whole stone fruit produced in California’s San Joaquin valley [37] (Table 1).

Experimental conditions used in the study are adapted from the food safety guidelines for fresh whole stone fruit produced in California’s San Joaquin valley [37] and based on discussions with the California Fresh Fruit Association.

## 2. Materials and Methods

### 2.1. Peaches and Nectarines

Unripened, unwaxed fruits (yellow flesh peaches—var. Autumn Flame and nectarines—var. August Fire) were procured immediately after harvest from Prima^®^ Wawona, Reedley, CA, USA. Upon receipt, fruits were visually inspected for defects (bruises, moldy growth and breaks in peel), and any defective fruit was discarded. All fruits were maintained at 4 °C, with 90% humidity, until use. A day before the experiment, the required number of fruits was transferred to room temperature (20 or 30 °C) for tempering prior to use [45,46].

### 2.2. Bacterial Cultures and Inoculum Preparation

A cocktail of *L. monocytogenes* isolates consisting of produce isolates (LM1, LM2, LM3—apple isolates) and human isolates (Scott A, LM 19115) was used for the study. Each isolate was cultured separately in 10 mL of sterile brain heart infusion broth (BHI) and Nalidixic acid (NA; 50 µg/mL) at 37 °C for 24 h with agitation (100 rpm). Cultures were then transferred for two consecutive 24-h periods onto brain/heart infusion agar plates containing NA (BHIN) to produce a bacterial lawn. To prepare the inoculum, growth from the bacterial lawn was transferred to 0.1% buffered peptone water (BPW) to an absorbance of 0.2% [47]. The approximate bacterial count in each culture was determined spectrophotometrically. Equal portions from each of the five isolates were combined to make the pathogen cocktail. The bacterial population in the *Listeria* cocktail was determined by plating 0.1 mL portions of appropriate dilutions on modified Oxford media with NA (MOXN; [48]), followed by incubation at 37 °C for 48 h [20]. Appropriate dilutions of the five-strain mixture in buffered peptone water (BPW) was used to obtain the desired level of inoculum. A high inoculum level (5 log CFU/fruit) was used to enable measurement of several log reductions in pathogens counts during the study [49]. Additionally, this study also incorporated a low level of inoculum (3 log CFU/fruit) in order to simulate low levels of pathogen contamination that are likely to occur under normal processing, storage and distribution conditions [50].

### 2.3. Fruit Inoculation

Fruits were individually spot-inoculated with the bacterial cocktail (7 or 5 log CFU/fruit) by placing 50 µL of the inoculum around the stem end. In order to prevent the inoculum from running off the side of the fruit, the inoculum was applied in small approximately equal volumes to 10 different locations [8,46,51]. After inoculation, fruits were held for 24 h at room temperature in a biosafety hood for the inoculum to dry. Staggered inoculation of the fruits was performed to maintain consistent drying time for all fruits [52]. Before each experiment, 15 fruits were sampled immediately following and after drying (24 h post-inoculation) to determine pathogen load and inoculum uniformity on the fruits [46,53].

### 2.4. Survival of Listeria on Peaches and Nectarines under Simulated Fruit Handling, Waxing and Storage Conditions

#### 2.4.1. Stone-Fruit Unloading and Staging Conditions at the Packinghouse (Temperature—18–20 or 28–30 °C (Ambient Cool and Warm Season Temperature), RH—40–50% (Ambient Humidity) and Holding Time—1 to 18 h)

This objective investigated the effect of stone-fruit handling at ambient temperatures and humidity as encountered during the staging and unloading of fruit at the packing facility. Following *Listeria* inoculation and drying, stone fruits were placed in unsealed sterile polycarbonate containers (8-3/4 in. ×8-5/16 in. ×8-3/4 in.; Fisher Scientific, Waltham, MA, USA) and stored at 18–20 (19 ± 1 °C) or 28–30 °C (29 ± 1 °C; ambient cool and warm season temperature), RH 40–50% (45 ± 5%; ambient humidity), for a time period of 1 to 18 h to simulate stone-fruit handling during transportation to and staging at the packing facility (Table 1). Relative humidity was monitored throughout the experiment, using a digital humidity/temperature/dew point meter (Traceable™, Fisher Scientific, Hampton, NH, USA). At designated times during the storage (0, 2, 6, 12 and 18 h), fruits were sampled for microbiological analysis, and the entire experiment was repeated three times. A similar experimental set up was used to evaluate *Listeria* survival on nectarines.

#### 2.4.2. Fruit Waxing (Mineral-Oil- and Vegetable-Oil-Based Fruit Finish) and Fungicide Application (Fludioxonil and Propiconazole) at the Stone Fruits Packing Facility (Temperature—18–20 or 28–30 °C (Ambient Cool and Warm Season Temperature), RH—40–50% (Ambient Humidity) and Holding Time—1 to 6 h)

Peaches/nectarines were inoculated as previously described and used to investigate the effect of mineral-oil-based (PrimaFresh^®^220) and vegetable-oil-based (PrimaFresh^®^55EU) fruit finish containing fungicides (fludioxonil (PacRite^®^FDL) or propiconazole (Mentor^®^)) on *Listeria* survival. The fruit finish and fungicides were kindly provided by Pace International. The fungicide/fruit finish formulations were prepared according to manufacturer’s instructions prior to application. Inoculated fruits were sprayed with one of four different wax/fungicide combinations, using a gravity-feed dual action air-nozzle sprayer at 28–30 °C (29 ± 1 °C) or 18–20 °C (19 ± 1 °C; ambient warm and cool season temperature) and 40–50% RH (45 ± 5%; ambient humidity; Table 1). The formulations included mineral oil + fludioxonil (MF), mineral oil + propiconazole (MP), vegetable oil + fludioxonil (VF) and vegetable oil + propiconazole (VP). Each fruit was sprayed with one pull each to the stem and calyx ends and three pulls to coat the rest of the fruit surface. Following waxing, a subset of fruits was sampled to ascertain pathogen load prior to storage. Then, fruits were packed in sterile boxes to simulate packinghouse practices and held under ambient warm and cool conditions as described above for up to 6 h. Surviving *Listeria* population on peaches was enumerated at different times during fruit holding. *Listeria* survival on nectarines was performed as described above.

#### 2.4.3. Refrigerated Storage of Waxed Fruit at the Packinghouse (Temperature—1–2 °C, RH 85–95% and Storage Time—3 to 4 weeks)

Following waxing, peaches and nectarines are hand-placed into the final containers, and these boxes are then cooled to 1–2 °C and placed in cold storage prior to being shipped to market in refrigerated trailers. Therefore, to investigate the effect of cooling and storage conditions on *Listeria* survival, peaches/nectarines inoculated with 7 log CFU/mL and 5 log CFU/mL of *Listeria* cocktail were allowed to dry. The four fungicide fruit-finish combinations were prepared and sprayed under warm ambient conditions, packed and held for 6 h, as per objective 2. Packed fruit were then stored at 1–2 °C (1.0 ± 0.5 °C) and RH of 85–95% (90 ± 5%) for four weeks (Table 1) to simulate fruit storage at the packinghouse. Surviving *Listeria* population on fruits were enumerated during the four-week storage period (0, 1, 5, 7, 14, 21 and 28 days). Similar experimental set up was employed to enumerate *Listeria* populations on nectarines

### 2.5. Microbiological Analyses

At each sampling time, stone fruits (*n* = 4) were individually transferred to sterile stomacher bags containing 100 mL of BPW. Each fruit was hand-rubbed for 2 min, and the BPW was analyzed for *Listeria* population and/or presence (enrichment). Briefly, BPW from the stomacher bags containing peaches or nectarines was serially diluted in BPW and duplicate 0.1 mL aliquots of the appropriate dilutions were surface plated on MOXN followed by incubation at 37 °C for 48 h [20,54]. In addition to enumeration, BPW samples were enriched in *Listeria* enrichment broth UVM at 35 °C for 24 h. A 1 mL aliquot of the enriched UVM culture was then added to 9 mL of Fraser broth and incubated at 35 °C for 24 h. When counts for the respective samples were negative by direct plating, enrichment broths were streaked on MOXN and incubated at 37 °C for 48 h. Presumptive colonies isolated from MOXN plates were confirmed as *Listeria monocytogenes*, using the Singlepath^®^ L’mono agglutination assay.

### 2.6. Statistical Analysis

Four fruits were sampled at each sampling time, and three independent trials were conducted. Pooled samples (*n* = 12 fruit/temperature/time point/variety under each experiment) were averaged, and the data were analyzed by using the mixed procedure of SAS ver. 9.2. Differences among the means were detected at *p* < 0.05, using the Fisher’s least significance difference test with appropriate corrections for multiple comparisons. Independent experiments were conducted to determine the effect of stone-fruit packinghouse conditions and practices on *Listeria* survival on peaches (var. Autumn Flame) and nectarines (August Fire).

## 3. Results

The results of our study did not demonstrate any statistically significant difference (*p* > 0.05) in the survival of *Listeria* on yellow-flesh peaches (var. Autumn Flame) and nectarines (var. August Fire) under simulated stone-fruit packinghouse conditions. Hence, only data for peaches are presented here.

### 3.1. Survival of Listeria on Peaches under Simulated Packinghouse Conditions

Approximately 6.60 ± 0.03 and 5.46 ± 0.05 log CFU of *Listeria* was recovered from the peaches inoculated with high and low inoculum levels, respectively, immediately following inoculation. Following the 24 h inoculum drying period, approximately 5.15 ± 0.06 and 3.25 ± 0.05 log CFU of the pathogen was recovered from the peaches at both inoculation levels. Similar results were obtained with nectarines. These results reveal that *Listeria* can survive in significant numbers on peaches and nectarines during the extended drying period. This surviving population would be representative of bacterial cells that can withstand desiccation on the fruit surface [46,55]. Hence, for all objectives, fruits were dried for 24 h following surface inoculation.

### 3.2. Effect of Fruit Unloading and Staging Conditions on Listeria Survival on Stone Fruits

**Figure 1:**
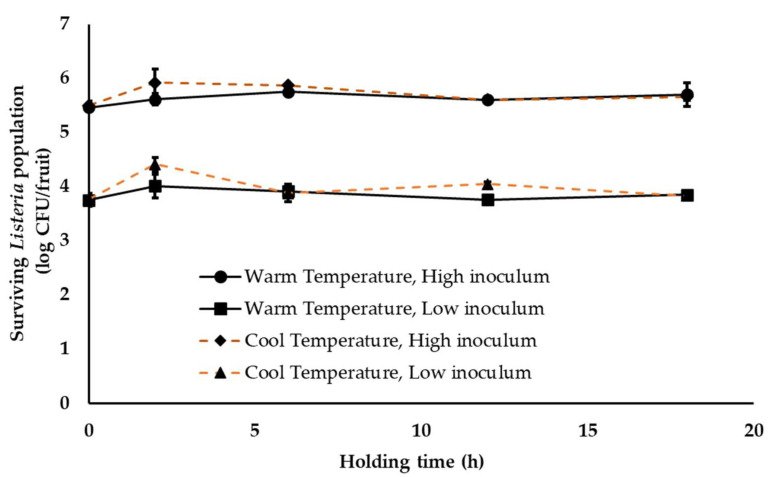
*Listeria* survival on peaches under simulated stone-fruit packinghouse unloading and staging conditions (temperature—18–20 or 28–30 °C (ambient cool and warm season temperature), RH—40–50% (ambient humidity) and holding time—1 to 18 h) at high (~5 log CFU/fruit) and low (~3 log CFU/fruit) pathogen load. Data are represented as the mean ± SE.

Following harvest, fruits are transferred to the packinghouse. At the packinghouse, stone fruits are either left in the transport trucks for unloading or are unloaded and staged in an unloading area. Fruits can be staged for 1–18 h before they are dumped into the packing line. Therefore, to simulate this initial fruit holding conditions, inoculated fruit were held at either 28–30 °C (29 ± 1 °C) or 18–20 °C (19 ± 1 °C) and RH 40–50% (45 ± 5%) for 18 h. As can be seen from Figure 1, survival of *Listeria* on peaches was not significantly affected by simulated stone-fruit unloading and staging conditions (*p* > 0.05). For instance, under warm ambient conditions, approximately 5.46 ± 0.05 and 5.71 ± 0.22 and 3.75 ± 0.12 and 3.85 ± 0.05 log CFU of the pathogen was recovered from fruit sampled at 0 and 18 h of the study under high and low pathogen inoculation levels, respectively (Figure 1). Under cool ambient temperatures, no significant change in *Listeria* population was observed throughout the study with ~5.66 ± 0.07 and 3.83 ± 0.04 log CFU of *Listeria* recovered from the peaches at 18 h at both inoculation levels, respectively (Figure 1). Although an increase in pathogen populations was observed at 18 h of storage, these results were not found to significantly different from *Listeria* counts at 0 h (Figure 1). Similar results were obtained with experiments performed on nectarines. Overall, irrespective of the ambient conditions and inoculum load, *Listeria* was able to survive equally well on peaches and nectarines. Further, although the simulated fruit unloading and staging conditions did not deter *Listeria* survival, they also did not favor any significant increase in pathogen population.

### 3.3. Effect of Fruit Waxing and Fungicide Application on Listeria Survival on Stone Fruits

**Figure 2:**
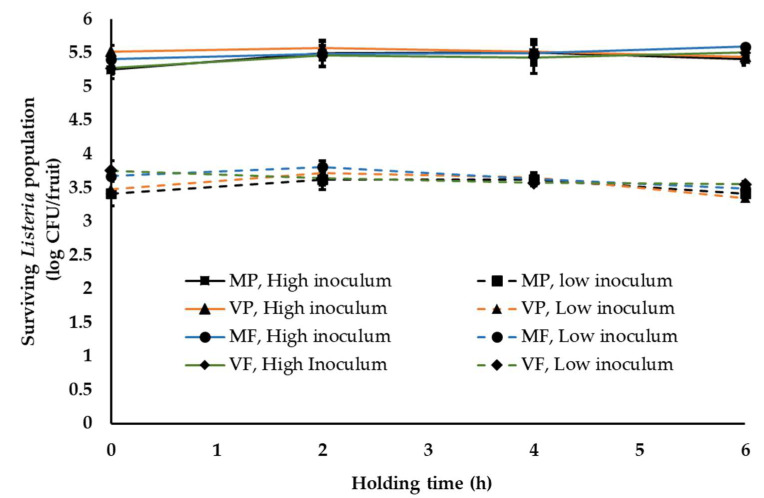
*Listeria* survival on peaches following fruit waxing and fungicide application under simulated packinghouse conditions (temperature—18–20 °C (ambient cool temperature), RH—40–50% (ambient humidity) and holding time—1 to 6 h) at high (~5 log CFU/fruit) and low (~3 log CFU/fruit) pathogen load. Fruit finish formulations used in the study include MP, mineral oil + propiconazole; VP, vegetable oil +propiconazole; MF, mineral oil + fludioxonil; and VF, vegetable oil + fludioxonil. Data are represented as the mean ± SE.

**Figure 3:**
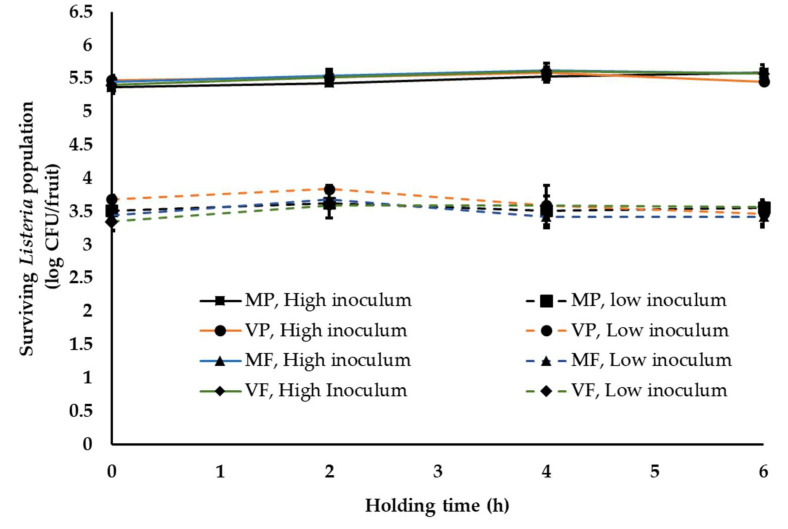
*Listeria* survival on peaches following fruit waxing and fungicide application under simulated packinghouse conditions (temperature—28–30 °C (ambient warm season temperature), RH—40–50% (ambient humidity) and holding time—1 to 6 h) at high (~5 log CFU/fruit) and low (~3 log CFU/fruit) pathogen load. Fruit finish formulations used in the study include MP, mineral oil + propiconazole; VP, vegetable oil + propiconazole; MF, mineral oil + fludioxonil; and VF, vegetable oil + fludioxonil. Data are represented as the mean ± SE.

One of the common practices at a stone-fruit packinghouse is application of fruit finish containing fungicides. This is generally applied once staged fruit are transferred to the packing line [37]. In order to simulate this waxing and fungicide application at the stone-fruit packinghouse, two commonly used fruit finish namely PF220 (mineral-oil-based, M) and PF55EU (vegetable-oil-based, V) were employed in combination with two widely used fungicides, propiconazole (Mentor^®^; P) and fludioxonil (PacRite^®^FDL; F). Following waxing, fruit are graded, sized, packed and moved to the cooling room. The average time between packing and cooling is generally about 4–6 h. During this time, fruits are held under ambient conditions. Hence, in this study, waxed fruit were packed in containers and held under ambient conditions for 6 h. Irrespective of the type of wax and fungicide applied, *Listeria* was able to survive in significant numbers on peaches (Figure 2 and Figure 3). At high inoculum levels and under cool ambient conditions, ~5.3 ± 0.14, 5.52 ± 0.1, 5.41 ± 0.1, 5.3 ± 0.1 and 5.40 ± 0.05, 5.44 ± 0.13, 5.59 ± 0.1 and 5.50 ± 0.12 log CFU of *Listeria* was recovered at 0 and 6 h from fruits treated with MP, VP, MF and VF, respectively (Figure 2). Further, no significant change in pathogen population was observed throughout the 6 h holding period. Similarly, at low inoculum levels, ~3.51 ± 0.09 and 3.45 ± 0.05 log CFU of *Listeria*/peach was recovered across different treatments at 0 and 6 h, respectively. Additionally, no significant reduction in pathogen population was observed on waxed fruits held under warm ambient conditions (Figure 3). Similar results were obtained with experiments performed on nectarines. As previously observed, regardless of the inoculation levels, stone-fruit waxing and fungicide application under ambient warm and cool temperatures did not favor *Listeria* growth, as evidenced from the absence of significant increase in pathogen population over time.

### 3.4. Effect of Refrigerated Storage Conditions on Listeria Survival on Waxed Stone Fruits

**Figure 4:**
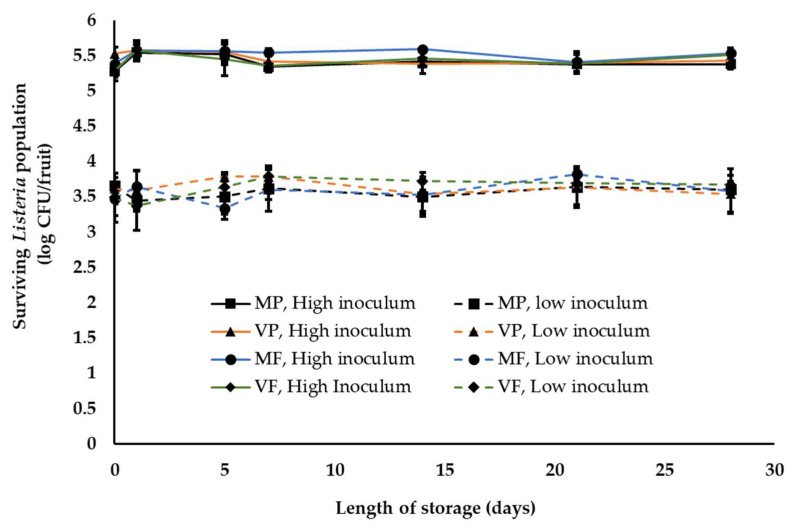
*Listeria* survival on peaches following fruit waxing and fungicide application and refrigerated storage under simulated packinghouse conditions (temperature—1–2 °C, RH—85–95% (ambient humidity) and length of storage—3 to 4 weeks) at high (~5 log CFU/fruit) and low (~3 log CFU/fruit) pathogen load. Fruit finish formulations used in the study include MP, mineral oil + propiconazole; VP, vegetable oil + propiconazole; MF, mineral oil + fludioxonil; and VF, vegetable oil + fludioxonil. Data are represented as the mean ± SE.

The final storage temperature and high humidity is particularly critical for peach and nectarines since they are highly perishable and deteriorate quickly at ambient temperatures [56,57]. Therefore, waxed fruit are held in cold storage for 2–28 days at 1–2 °C and RH—90–95% before being shipped to market in refrigerated trailers. Since we did not observe any significant difference in *Listeria* population on waxed fruits held at ambient warm and cool temperatures, fruits were waxed and packed under warm ambient conditions prior to refrigerated storage. Over the four-week storage period, irrespective of the inoculum load and wax/fungicide treatment, no significant reduction in *Listeria* population was observed on both peaches and nectarines. At the end of storage (4 weeks) approximately 5.3–5.5 and 3.5–3.6 log CFU of *Listeria* was recovered from peaches and nectarines at high and low inoculum level, respectively (Figure 4). This indicates that although low temperature may deter pathogen growth it does not inhibit *Listeria* survival on peaches and nectarines. Overall, ambient conditions and practices simulating the packing-house were not found to significantly deter *Listeria* survival on peaches and nectarines.

## 4. Discussion

Scenario analyses conducted by the FDA-CFSAN (Center for Food Safety and Nutrition) and USDA-FSIS (Food Safety and Inspection Service) demonstrated that risk for listeriosis from a given food was primarily governed by the food’s composition and its handling and storage conditions [58,59]. All of these factors are highly relevant to fresh produce and its processing environment. Further, the absence of practical technologies that provide a necessary kill step for pathogens on fresh produce, including peaches and nectarines, provides a unique challenge to the stone-fruit industry. Additionally, recall of stone fruits due to potential *Listeria* contamination has highlighted the need for generation of risk reduction knowledge towards the development of preventive controls for foodborne pathogens. In order to develop preventive controls, it is critical to understand the effect of stone-fruit processing conditions on pathogen survival. Hence, this study was performed to determine the influence of commercial packinghouse conditions and practices on *Listeria* survival on stone fruits.

In addition to the ambient environment, the inherent nature of the produce, including the surface structure and topographic characteristics, can influence pathogen attachment, survival and growth [32]. Therefore, to account for differences in produce type, *Listeria* survival was assessed on peaches and nectarines. Peaches in general have a downy or fuzzy surface while nectarines have smooth skins [60]. Nectarines arose as peach mutants, with differences in fruit size, shape, firmness, external color, aroma, flavor and disease resistance and better storage characteristics than peaches [61,62]. Given the topographical differences, Collignon and Korsten [8] studied pathogen attachment and survival as influenced by produce type. They demonstrated that survival and recovery of pathogens differed with different fruits. For instance, *Listeria* was found to survive better on peaches than on plums. Contrary to these results, De Jesus et al. [15] reported that stone-fruit type did not affect *Listeria* survival under refrigerated storage (4 °C, 90–92% RH) for 26 days. Similarly, we did not observe any significant difference in *Listeria* survival on yellow flesh peaches (var. Autumn Flame) and nectarines (var. August Fire; Figure 1, Figure 2, Figure 3 and Figure 4). Survival patterns of *Listeria* at both high and low inoculum did not show any significant differences between peaches and nectarines. This is in agreement with reports on the growth and survival of *L monocytogenes* in raw fruits and vegetables with varying surface characteristics [63,64,65,66].

In addition to the above factors, pathogen type also influences its survival on produce. Studies investigating *Salmonella*, *E. coli* O157 and *Listeria* survival on produce have demonstrated a difference in growth and survivability of the pathogens [67]. Different studies have demonstrated a longer persistence of *Listeria* on fresh produce at refrigeration temperatures when compared to the other pathogens [20,48,67]. Hence proper temperature regulation and monitoring is of particular significance in the control of *Listeria*. Several studies have investigated the persistence of *Listeria* on different produce, including tomatoes, asparagus, stone fruits, apples, strawberries and surface of fruits with inedible skin, such as bananas and watermelons, under simulated processing, storage and distribution conditions [8,34,50,68,69,70]. These studies demonstrated that *Listeria* could survive on fruit surface for extended periods of time, under different storage temperatures, including ambient temperature (21 °C), cold storage at 10 or 4 °C and freezing at −20 °C [8,34,67]. In particular, Collignon and Korsten [8] observed a significant reduction in *Listeria* populations on peaches and plums during the initial days of storage under simulated commercial export chain conditions. However, in the present study we did not observe any significant reduction in pathogen population across all storage conditions (Figure 1, Figure 2, Figure 3 and Figure 4). This could be due to differences in temperature-relative humidity and length of storage conditions employed in the present study.

Relative humidity also significantly influences pathogen survival on fruits. Likotrafiti et al. [35] demonstrated that *Listeria* survival on fresh lettuce and cucumber was unaffected by change in relative humidity (RH). Equal populations of *Listeria* were recovered from produce maintained at an RH of 55 or 90%. However, *Listeria* growth was found to be slower at lower RH. Similar study performed by Palumbo and Williams [71] evaluating *Listeria* survival in different menstrua demonstrated that reduced growth rate at lower RH (59%) was associated with prolonged survival and recovery of *Listeria*. The results from these studies highlight the unique ability of *Listeria* to survive and withstand reduced RH or dry conditions that are commonly encountered on produce surface. This finding is in agreement with our study where warm summer temperature of 28 to 30 °C and ambient cool temperature of 18 to 20 °C and 40–50% RH, did not have any significant reduction on the inoculated *Listeria* population, both at high and low inoculum (Figure 1, Figure 2 and Figure 3). Similarly, fruit storage under conditions of high relative humidity (85–95%) also did not affect pathogen survival (Figure 4). Hence, irrespective of the ambient relative humidity, *Listeria* was able to survive in significant numbers on stone fruits. However, although these conditions supported *Listeria* survival, they did not promote pathogen growth on the produce.

Besides the ambient conditions associated with stone-fruit processing, fruit waxing and fungicide application are an integral part of the packinghouse practices. In the case of stone fruits, besides helping to reduce moisture loss, fruit finish is used as a carrier for fungicides [37,72,73]. Commercial fruit finish widely employed in the industry are either mineral or vegetable oil based. In addition to waxing, stone fruits are treated with fungicides for post-harvest control of brown rot and sour rot [40]. The commonly used fungicides in the stone-fruit industry include propiconazole and fludioxonil. Hence to simulate industry practices, a combination of fruit finish and fungicides were employed in this study. As with other practices, wax treatments could influence pathogen survival by providing limited dehydration protection to the microbe and thereby favoring bacterial survival [39]. Similarly, several studies have demonstrated the effect of fungicide application on bacteria survival. Sethi et al. [41] observed that fungicide application enhanced the growth of *E. coli*, *Bacillus subtilis*, *Serratia marcescens* and *Enterobacter*. Further studies demonstrated that different fungicides exert differential effects on bacterial survival. Yen et al. [42] demonstrated that the application of propiconazole inhibited microbial population, while triadimefon induced bacterial survival. This variation in effect could be explained by the difference in mode of action of the fungicides [43]. These studies indicate that post-harvest practices, including the application of fruit finish and fungicide, can influence *Listeria* survival and growth on fruit surface. Along the same lines, Kenney and Beuchat [73] evaluated the effect of six different wax formulations, including Carnauba Gold, on *Salmonella* Muenchen survival on apples. They observed that waxing by itself did not result in any significant reduction in *Salmonella* populations on apples. Further, our previous study evaluating *Salmonella* survival on mangoes did not observe any effect of waxing on pathogen survival [46]. Similarly, in the present study, *Listeria* survival was unaffected by the inoculum level, the type of wax and fungicide used and holding/storage time (Figure 2, Figure 3 and Figure 4). More specifically, no significant reduction in pathogen population was observed under conditions simulating commercial stone-fruit handling and storage.

## 5. Conclusions

The results of our study demonstrate that current stone-fruit handling conditions do not favor *Listeria* growth. However, once contaminated, *Listeria* can survive on the fruit surface in significant numbers under current packinghouse conditions and practices. Further, commercial waxes and fungicides employed in the stone-fruit industry did not exhibit any inhibitory effect on pathogen survival. Therefore, once contaminated, fruit can serve as a potential source for *Listeria* transmission along the post-harvest environment. In conclusion, these results demonstrate the need for development and implementation of preventive controls at the stone-fruit packinghouse to prevent *Listeria* contamination and deter pathogen persistence.

## Figures and Tables

**Table 1:** Stone-fruit handling, processing and storage conditions at a commercial packinghouse.

Stage	Holding Temperature (°C)	Relative Humidity (%)	Holding/Storage Time
Unloading and staging			
Warm season	28–30	40–50	1–18 h
Cool season	18–20	40–50	1–18 h
Fruit finish and fungicide application **^ǂ^**			
Warm season	28–30	40–50	1–6 h
Cool season	18–20	40–50	1–6 h
Refrigerated storage	1–2	85–95	2–28 d

^ǂ^ Fruit finish/fungicide combinations used include mineral-oil- or vegetable-oil-based fruit finish containing fungicides fludioxonil or propiconazole.

## Data Availability

Data from this study were submitted as project results and performance reports to CPS and can be accessed at https://www.centerforproducesafety.org/amass/documents/researchproject/403/CPS%20Final%20Report_Amalaradjou%2C%20January%202018.pdf (accessed on 8 August 2021).
